# A 1-Diopter Vertical Prism Induces a Decrease of Head Rotation: A Pilot Investigation

**DOI:** 10.3389/fneur.2016.00062

**Published:** 2016-04-28

**Authors:** Eric Matheron, Ava Zandi, Danping Wang, Zoï Kapoula

**Affiliations:** ^1^IRIS Team, Physiopathologie de la Vision et Motricité Binoculaire, CNRS FR3636, UFR Biomédicale, Université Paris Descartes, Paris, France; ^2^CNRS FR3636, UFR Biomédicale, Université Paris Descartes, Paris, France

**Keywords:** prism, heterophoria, head rotation, spine, neck, motor control, upright stance, three-dimensional kinematics

## Abstract

Clinical studies in non-specific chronic arthralgia and back pain seem to indicate an association between vertical heterophoria (VH – latent vertical retinal misalignment) and asymmetrical head rotation. Such clinical observations suggest a link between VH and head rotation, but this was never tested. The purpose of this study was to simulate a VH in healthy subjects and examine its influence on the amplitude of active head rotation during 3D motion capture in upright stance. Subjects were asked to rotate their head three times from the straight ahead position and then to the right, back to straight ahead, to the left, and back to the straight ahead again. Three randomized conditions were run: normal viewing, with a 1-diopter prism base down on the dominant eye, or the non-dominant eye. The most important finding is that the experimental VH whichever the eye with the prism induces a significant decrease in the mean angle of head rotation compared to the normal viewing condition. This decrease was significant for rotation to the left. We suggest that the prism-induced VH modifies the reference posture and thereby affects head rotation; further studies are needed to confirm this effect and to extend to other types of dynamic activities.

## Introduction

Motor coordination between the head movement, gaze direction, and the rest of the body is constant in daily life, whatever the motor efficiency, automatic, reflex, or intentional. In an intentional way, gaze orientation accompanied by the rotation of the head on the trunk, i.e., the neck or the cervical spine rotation in the horizontal plane is common, for instance, in a sitting position when we are watching a tennis match or in upright stance watching a soccer match aiming to follow the ball. Similarly, when we stop because we want to cross a street, we look right and left to see if cars are coming or not. Therefore, this act is done with or without a visual target.

For these tasks, postural control is required. Maintenance of postural balance during quiet upright stance is complex and requires processing of signals from the visual, vestibular, and somatosensory systems (1). The central nervous system (CNS) performs coordinated transformations of these signals and permanently generates adapted muscular response as corrective torque through the action of a feedback control system [e.g., Ref. (2–4)]. Postural control is involved in the control of body segment orientation and body stabilization, which is a prerequisite for perception and action (5), and is the basis for body stability during dynamic activities (e.g., motor actions such as voluntary movements) (6–8).

Vertical heterophoria (VH) is the vertical misalignment of the eyes when the binocular vision is briefly interrupted and vertical orthophoria (VO) when there is no misalignment (9, 10). VH can exist in healthy subjects, inferior to 1 diopter, on average 0.16 ± 0.01° corresponding to 0.28 diopter (11, 12). Amos and Rutstein (9), and Scheiman and Wick (13) reported that subjects with VH could present various complaints, such as neck pain, where VH could be induced by eye refraction problems as astigmatic or hypermetropia. In the absence of neuropathy or rheumatism, clinical studies indicate an association between non-specific chronic pain, VH, qualitative balance control, and spine and peripheral joints mobility that were clinically evaluated (14–16).

In non-specific chronic pain, VH is present and is <1 diopter (17, 18). It was reported that in patients with non-specific chronic pain associated with VH, a specific proprioceptive physiotherapy acting on oropharynx, temporomandibular joint, and/or pelvis most of the time restored VO immediately (14), diminished pain [evaluated with a subjective visual analog scale (VAS) (19, 20)], normalized behavior in the balance tests, improved mobility of peripheral joints and of the spine including the neck after initial alternation, but this remains to be precisely measured (15, 16). To test these clinical reports, a controlled experiment was run in healthy young adult subjects with or without simulated VH by a 2-diopter vertical prism base down on the dominant eye or on the non-dominant eye on postural control in terms of stability during quiet upright stance (21). The prism modified the base situation which accentuated or reduced the natural VH. The subjects were looking at a target at a close (40 cm) or at a far (200 cm) distance, and they were able to fuse the targets. Postural stability decreased whatever the viewing distance when the prism was on the non-dominant eye; when the prism was on the dominant eye, stability improved but only while looking at the far target. The use of small prisms was already known to induce postural behavior change in healthy subjects (22). It is the deviation of the luminous ray by a prism as visual input, which is at the origin of the difference in relationship between extraocular proprioception, eye alignment, and vision.

The oculomotor response induced by such prisms (vertical vergence) is slow (a few seconds), it can be asymmetric between the two eyes, and the magnitude of the response can fluctuate to be higher or lower relative to the required value of the prism (23–27). Therefore, there is a modification of the eye position in the orbit and so on the extraocular muscles proprioceptive ambiance that act on postural control, and on the tonic efficiency hypothetically up to the level of the extensor muscles of the lower limbs (28).

In clinical physical examination in non-specific back pain, the head rotation without movement of the trunk in the horizontal plane is commonly used; frequently head rotation appears limited in terms of amplitude on one side [e.g., Ref. (29–31)].

In order, to test this hypothesis in the present study, we induce VH with a prism in healthy subjects, and we test the amplitude of voluntary head rotation in upright stance. We hypothesize a central link between VH and head rotation.

## Materials and Methods

Eight healthy young subjects (three females and five males) in the range 16–32 (24 ± 6.5) years old participated in this study. They had no neurological, ophthalmological, or musculoskeletal symptoms or troubles (problems) and were not under any medication. They did not wear eye glasses.

Eye dominancy was determined using the hole-in-card test (32). Holding a card with a hole in the middle with both hands, subjects were asked to look at a target through the hole and close each eye separately: the eye which sees the target is the dominant eye, as when they move the card toward his/her face.

Detection and measurement of VH was done with the Maddox Rod Test combined with an appropriate prism value for each eye, which was <0.57°, thus in the physiological range (11). This test is one of the most appropriate (33).

**Figure 1:**
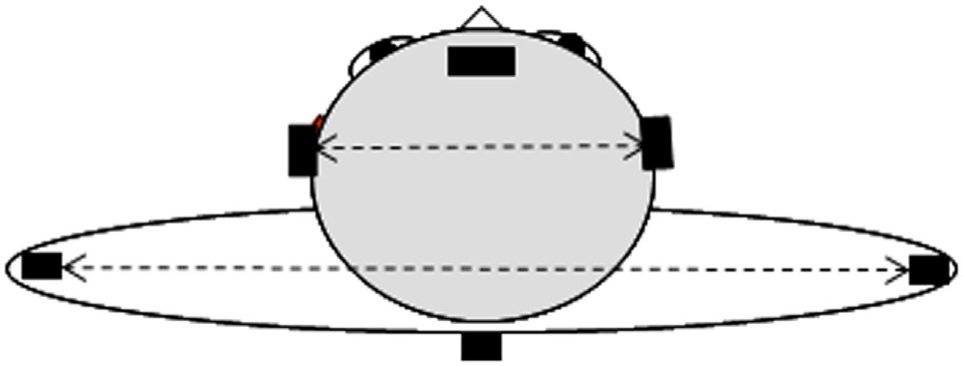
**Codamotion markers: three placed on the head on a head band – one in the middle of the forehead and two each side of the head above the ears; three placed at the shoulder girdle – one on the spinous process of the first thoracic vertebra and two on the left and right acromions**.

Subjects were asked to stay in the middle of an experimental room in quiet upright stance, barefoot and keeping their arms along their body. The subjects wore a special spectacle upon which one could easily put on a 1-diopter vertical prism base down if the case. Amplitude of rotation has been determined, while subjects were asked to rotate their head to the right, then to the left alternately three times without forcing the movement, each time with a stop at the center (i.e., in a straight ahead position), the shoulder girdle stable. Head rotation on the trunk was measured with the CODAMOTION active markers (Version 6.70.16-CX1-CodaSharc V3.02, Charnwood Dynamics Ltd., Rothley, UK) placed on the head and at the shoulder girdle level for 3-D recording (see Figure 1), and the analysis was done *via* Matlab software at the faculty of medicine of Paris Descartes. Three randomized conditions were run: in normal viewing, with a 1-diopter vertical prism base down on the dominant eye or on the non-dominant eye. Three minutes of rest time have been considered between the trials. Before the first recorded trial, subjects were asked to repeat an alternate rotation of their head three times to avoid the tixotropia effect [i.e., muscle warm (34, 35)]. Considering one vector between the two markers located on the shoulders and another vector between the two markers located on both sides of the head, the changes in the angle between these two vectors has been plotted in XY plane (the projection of the vectors on the ground). For all conditions, an offset has been considered to keep the starting point of all of the plots at 0, in order to make the maximum and the minimum points of all of these plots comparable. Mean angle of three times, for the maximum rotation to the right, and the maximum rotation to the left, have been calculated. Therefore, for each subject, six measures were carried out (right and left in normal viewing, right and left with the prism on the dominant eye, and right and left with the prism on the non-dominant eye). According to the number of subjects (eight), Anova Frideman for non-parametric statistics have been applied to the values [χ^2^ (6 − 1 = 5 degrees of freedom)], and *p* < 0.05 was considered significant. Then, as *p* < 0.05, the Wilcoxon-matched paired test has been applied to localize significant changes, with the level of significance always set at *p* < 0.05. Analysis has been done for two different classifications: position of the prism according to the eye dominancy (i.e., dominant and non-dominant eye) and position of the prism according to the direction of the head rotation (i.e., right and left eye). Data analysis was performed using STATISTICA software version 7.1.

The investigation adhered to the tenets of the Declaration of Helsinki and was approved by the Institutional human experimentation committee, the “Comité de Protection des Personnes” (CPP) Ile de France VI (No: 07035), Necker Hospital in Paris. Written informed consent was obtained for all subjects after the nature of the procedure had been explained.

## Results

**Figure 2:**
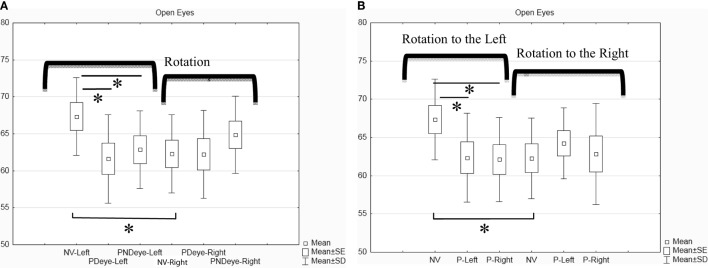
**Box and whisker plot for open eyes condition (in degrees)**. **(A)** Classification of the rotation according to the eye dominancy. **(B)** Classification of the rotation according to the position of the prism (left or right eye). Asterisk indicates significant difference (*p* < 0.05).

The rotation has been separately analyzed to the right and to the left for each of the three conditions (normal vision, prism on the dominant or non-dominant eye). The mean of the angle of rotation for the eight subjects for each condition is shown in Figure 2A. The Friedman ANOVA test showed a significant effect on the amplitude of head rotation [*F*_(5,8)_ = 12.57; *p* = 0.027]. The Wilcoxon-matched pair test showed the following significant differences: (1) the amplitude of rotation to the left in normal vision is significantly higher than the amplitude of rotation to the right in normal vision (*z* = 1.96; *p* = 0.049); (2) the amplitude of the rotation to the left with the prism on the dominant eye is significantly lower than in normal vision (*z* = 2.52; *p* = 0.011); and (3) the amplitude of the rotation to the left with the prism on the non-dominant eye is lower than in normal vision (*z* = 2.52; *p* = 0.011). This decrease with prism is even stronger when the prism is on the dominant eye. We obtained similar results when we compared the conditions according to left versus right eye (see Figure 2B).

## Discussion

### Summary of the Results

The main result is the decrease in the amplitude of head rotation to the left with the prism, no matter which is the dominant or non-dominant left or right eye wearing the prism. This result shows for the first time the impact of an experimentally induced VH by a 1-diopter vertical prism base down on eye-head rotation. The other important observation is the existence of a baseline difference in the amplitude of head rotation, which was greater to the left than to the right even without any prism. These results will be discussed below.

### Sensory Activation of the Prism

The prism used was of a weak power and created a small disparity between the two retinal images (0.57°). Such disparity could be compensated by the sensory visual system. Despite the misalignment of the two images received by each eye, fusion was still possible, as the prism would cause an adapted increase in the Panum’s fusion (36). If there were such a sensory adaptation *via* the enlargement of the Panum’s fusion area, one should not observe modification of head rotation. Thus, our results do not favor the interpretation in terms of sensory activation alone.

This small vertical disparity induced by the prism provides a visual error signal presumably integrated in the vestibular nuclei, which are known to be located at the base of the spinal motor neurons and oculomotor efferents (37, 38). So the basic muscle tone of the body before starting the voluntary head rotation could have been modified. This agrees with previous reports, for instance: by Magnus (39) who explained that the visual perception of our surrounding space intervenes in the regulation of the postural tonus; Gagey (28) reported a modification of the tone repartition down to lower limbs induced by a small prism in front of one eye; Séverac et al. (40) also reported different postural responses to prisms.

### Motor Activation of the Prism

This prism causes binocular disparity, providing a visual signal to the CNS that could stimulate compensatory eye movements, and thus modifying the extra oculomotor muscle proprioception. The eye with a 2-diopter prism is moving in the direction of the prism and the opposite eye (i.e., without prism) also moves in the opposite direction before stabilizing (27, 41). In such case, in addition to proprioceptive signals of extraocular muscles, there are central efferent signals of motor commands sent to the occulomotor system. Because of known synergy between eye movement commands and neck muscle activity (42), the eye movements elicited by the prism could lead to the activation of neck muscles involved in the head rotation. Zetterberg et al. (43) demonstrated the link between eye lens accommodation and trapezius muscle activity during dynamic near–far eye movement visual tasks. In our study, the prism modified mostly vergence and not accommodation, although cross couplings between vergence and accommodation are known to exist (44). Earlier, Bexander and Hodges (45) examined the neck muscle activity in controls and in subjects with whiplash-associated disorders and demonstrated a modified cervico-ocular coordination for the latter. Even earlier, Bexander et al. (46) recorded electromyographic activity of neck muscles both intramuscularly and with surface electrodes. They showed that the orientation of the eyes, relative to the orientation of the head, modified the discharge of the neck muscles: less discharge when the eyes were maintained in an orbital position opposite to the direction of the head. All these studies are relevant for the present one, which is the first to deal with a vertical eye misalignment in healthy adults induced by a vertical prism.

Thus, our results could be understood in this context: the 1-diopter vertical prism activates eye movement responses that interfere with head rotation. A limitation of this study was the absence of objective eye movement recording; another study (27) using eye movement recordings showed that a small vertical prism (2-diopter) induced a vertical vergence, the amplitude of which depended on eye dominance.

### No Prism Effect on Right Head Rotation

The question here is why there is no significant limitation of head rotation to the right while having the prism. Keeping up with the two interpretations mentioned above, sensory and motor, no effect on the rotation to the right could imply that sensory fusion and the required compensatory eye movement were well achieved for such rotation. Consequently, turning the head to the right would be less influenced by the misalignment of the image created by the prism.

Another explanation could be related to the fact that without the prism, i.e., in normal vision, the mean maximum range for the left rotation was greater than for the right rotation; the prism could have a lower impact on the right side (the effect being somehow proportional, the larger the amplitude of the initial rotation the more visible the prism effect would be). Some healthy subjects recruited had a VH <1 diopter (5/8); it is possible that some subjects, from the beginning, had a significant asymmetry between the two sides. Further studies with a larger population are needed to establish the difference and understand better its origin.

Another reason could be the fact that head rotation was always starting from straight ahead to the right, followed by straight ahead to the left; perhaps the effect of the prism is taking place later on. This is a limitation of the present study. However, to try to minimize this possibility, before recording, subjects turned their head alternately three times to avoid tixotropia effects (34, 35).

Finally, one can think that the eye dominancy could have an effect on head rotation reduction by the prism; more than the half of our subjects (five out of eight) were left eye dominant. Looking at the difference in our data between the maximum angle of the head rotation to the left side in normal vision, and the maximum angle of the rotation to the same side while having the prism in front of the left eye, the five subjects with the dominant left eye showed the higher difference. In contrast, when we looked at the difference for the rotation to the right side with and without the prism on the right eye, we did not see a difference. This could be because there were only three subjects with a dominant right eye. In perspective, here as well, there is the necessity to increase the number of the included subjects according to their eye dominancy.

## Conclusion

The present prospective preliminary study has clinical and theoretical relevancies, providing for the first time, to our knowledge, indication for a synergy between vertical eye alignment and head rotation. The effect of the prism reveals an existing synergy between extraocular muscles and neck muscles; the disparity induced by the prism leads to a change in extraocular and neck muscle activity, and in the proprioceptive ambiance. Further EMG studies would be of interest to identify the muscle changes involved in head rotation (left and right splenius capitis, and left and right sternocleidomastoid muscles) comparing head rotations with and without prisms.

The limitation of the study on small number of subjects performing the same sequence of head rotation (straight ahead to right − straight ahead to left), and also the absence of simultaneous eye movement and neck EMG recordings, means that further research to bridge such gaps would be of theoretical and clinical interest.

## Author Contributions

EM: conception and design of the work; acquisition, analysis, and interpretation of data; drafting the work; and final approval of work and its integrity. AZ: acquisition, analysis, and interpretation of data; drafting the work; and final approval of work and its integrity. DW: design of the work; acquisition, analysis, and interpretation of data; and revision and final approval of work and its integrity. ZK: conception of the work; analysis and interpretation of data; drafting the work; and revision and final approval of work and its integrity.

## Conflict of Interest Statement

The authors declare that the research was conducted in the absence of any commercial or financial relationships that could be construed as a potential conflict of interest.
